# Hypodermic Injection of Eucain

**Published:** 1900-06-15

**Authors:** W. L. Overholser

**Affiliations:** Winamac, Ind.


### HYPODERMIC INJECTION OF EUCAIN.

BY W. L. OVERHOLSER, WINAMAC, IND.

On Thursday, October 12th, I injected about twenty minims of a 4 percent solution of eucain B around three teeth, the upper left first bicuspid and both lower third molars. Two of them I extracted, but the right lower third molar I broke off, leaving the roots in.

The next morning there was some swelling, which continued to grow worse, and by noon became quite marked. It continued to get worse until on Monday, four days after the operation, it became quite alarming and the family physician was sent for. He described the condition he found as follows: The tongue, sublingual and glossus muscles were swollen to such an extent that the tongue was pushed out of the mouth one-third its length, and respiration was so much impeded by the enormous swelling of the glands that it seemed for a few hours that nothing short of tracheotomy would save the patient's life. However, the following day the thickened mucous membrane lining the mouth and fauces as far down the throat as the pharynx sloughed off, leaving the parts bare and bleeding. At this stage the swelling of the glands began to disappear, and respiration became more easy. In about ten days from the commencement of the attack the patient recovered. The swelling was more marked on the right side.

The patient was a farmer's wife, twenty-four years old, and she did not take any care of her teeth.

Do you think it was caused by the eucain? If not, what was the cause?—*Dental Cosmos.*

